# The Effect of Substrate on Water Quality in Ornamental Fish Tanks

**DOI:** 10.3390/ani12192679

**Published:** 2022-10-05

**Authors:** Myriam Vanderzwalmen, Daniel Sánchez Lacalle, Priyadarshini Tamilselvan, Jason McNeill, Dorine Delieuvin, Khadidja Behlouli, Andrew Hursthouse, Iain McLellan, Mhairi E. Alexander, Fiona L. Henriquez, Donna Snellgrove, Katherine A. Sloman

**Affiliations:** 1 Institute of Biomedical and Environmental Health Research, University of the West of Scotland, Paisley Campus, Paisley PA1 2BE, UK; 2 Institute of Biomedical and Environmental Health Research, University of the West of Scotland, Lanarkshire Campus, Glasgow G72 0LH, UK; 3 WALTHAM Petcare Science Institute, Freeby Lane, Waltham-on-the-Wolds, Leicestershire LE14 4RT, UK

**Keywords:** environmental enrichment, gravel substrate, home aquaria, ornamental fishes, sand substrate, substrate enrichment

## Abstract

**Simple Summary:**

Fish kept as pets are almost always held in tanks with substrate such as gravel or sand on the bottom of the tank. This may be added as a form of enrichment to encourage natural fish behaviours, or for aesthetic reasons. However, substrate can also harbour elevated levels of waste products and unwanted bacteria; therefore, whether the use of substrate in home aquaria is advantageous or disadvantageous has not been fully considered. Here, we investigated whether there was a difference in water quality in home aquaria that contained either no substrate (bare tanks), plastic plants as enrichment but no substrate, sand or gravel substrate. Water quality (e.g., temperature, oxygen, pH and ammonia) and the presence of bacteria were measured over a 7-week period. As water quality can also vary with the season, the study was repeated at different times of the year. Addition of both gravel and sand substrate resulted in increased pH and the waste products ammonia and nitrate. Substrate was also associated with a greater presence of bacteria. In conclusion, the use of substrate affected water quality, with further research needed on the use of substrate in home aquaria.

**Abstract:**

Almost all home aquaria contain substrate, either as intentional enrichment or for aesthetic purposes. For fishes, benefits of structural enrichment have been well considered, particularly in research and aquaculture settings. However, our understanding of the impacts of tank substrate as enrichment is limited. While substrate can induce foraging in some species, a major drawback is the potential of substrate to harbour elevated levels of waste and pathogenic bacteria. Here, we considered whether substrate as a form of environmental enrichment significantly altered water quality and bacterial presence in home aquaria. Water quality (temperature, oxygen, pH, TAN, unionised ammonia, nitrate, Ca^2+^, Na^+^, Mg^2+^ and K^+^) and bacterial presence (*Pseudomonas* spp.) were measured over two seven-week periods in stand-alone, tropical, freshwater tanks that simulated home aquaria. The following four enrichment conditions were considered: bare tanks, plastic plants, gravel substrate or sand substrate. The addition of both gravel and sand resulted in increased pH, concentrations of total ammonia nitrogen and nitrate. Substrate was also associated with a greater *Pseudomonas* presence. Decreased pH alongside an increased concentration of ions were also observed depending on the time of year. In conclusion, enrichment type affected the water quality of home aquaria, with further research needed on the role of the tank biome in fish welfare.

## 1. Introduction

Provision of environmental enrichment for fish aquaria is generally considered to have positive impacts on fish welfare [1,2,3,4], with the use of physical enrichment (i.e., structural or substrate enrichment [5]) recommended for home aquaria. Structural enrichment can include shelters or areas of real or artificial vegetation, which provide refuge from intra- and interspecific aggression. In some fish species, the addition of structural enrichment can affect behavioural development [6], induce physiological benefits [7,8,9] and aid recovery from stressful situations [10]. Another type of structural enrichment almost always found in home aquaria is the addition of substrate, such as sand or gravel, which has been shown to increase foraging behaviour in goldfish *Carassius auratus* [11]. Benefits of substrate enrichment have also been noted in gilthead seabream *Sparus aurata* [12] and flatfishes [13,14,15], but substrate as a form of enrichment is less common in fish research facilities [16].

Substrate as a form of enrichment for fish aquaria has received less attention than other physical forms of enrichment [1,17]. One of the major drawbacks to the use of substrate, and indeed other physical structures to a lesser extent, is the potential for pathogenic bacteria and fungi to grow on the increased surface area. The limited research in this area has shown that the addition of substrate can elevate levels of nitrogenous waste due to inefficient waste removal [12] and increase bacterial load compared to sterile barren environments [18]. While it is essential to maintain colonies of beneficial microbes in aquaria to facilitate breakdown of nitrogenous wastes, pathogenic microbes need to be kept under control and routine practices, such as water changes, can alter microbial load diversity [19]. The attachment of bacteria and other microbes to surfaces can be influenced by several factors, including pH, nutrient level, temperature and water quality. Electrostatic and hydrophobic interactions of the surface will also influence bacterial attachment. For example, a marine *Pseudomonas* spp. attached in large numbers to hydrophobic plastics with a low surface charge, in moderate numbers to hydrophilic metals with a positive or neutral surface charge and in very low numbers to hydrophilic materials that possessed a negative charge [20]. Therefore, microbes in aquaria will attach to substrates and other forms of environmental enrichment, depending on the materials they are made from.

Prevalence of potential bacterial pathogens in aquarium water that can cause disease in both fishes and humans has been previously documented [21,22]. Pathogenic microbes may be introduced into home aquaria through tap water or through the addition of new items to the tank, including environmental enrichment, live food and new fishes [22,23]. *Pseudomonas* spp., and *Aeromonas* spp., Gram-negative, opportunistic pathogens responsible for systemic infections in fishes, have been most predominantly encountered [24]. Other widespread aquatic pathogens known to cause disease in fishes include *Mycobacterium* spp. [22,25] and *Acanthamoeba* spp. [26]. Tap water used for most home aquaria may be supplied from surface or ground water and is usually pre-treated. However, impurities associated with tap water are inevitable, originating from either the water source itself, water treatment processes [27], or the pipes through which it is supplied [28,29]. Quality of domestic water is also likely to change on a seasonal basis through hydrological processes that route precipitation [30]; thus, seasonal impacts on home aquaria water quality are possible.

There has been a growing interest among the scientific and hobbyist community to understand the potential advantages and disadvantages of substrates as environmental enrichment in relation to bacterial load and water quality [12,18]. The potential for increased surface area provided by other types of structural enrichment to influence bacterial growth also remains unknown. The aim of the present study was to determine whether environmental enrichment significantly altered water quality and bacterial presence in a home aquarium setting. Changes in water quality (temperature, oxygen, pH, ammonia, Ca^2+^, Na^+^, Mg^2+^ and K^+^) and bacterial presence (*Pseudomonas* spp. as an indicative freshwater pathogen) were compared between environmental enrichments (bare tanks, gravel only, sand only and plastic plants only).

## 2. Materials and Methods

Ornamental fish tanks were set up as stand-alone systems at the University of the West of Scotland, mimicking typical home aquaria. For this, 20 glass tanks (25 L) were filled with 20 L of charcoal filtered tap water. Each tank contained an internal filter (AllPondSolutions, Uxbridge, UK 200 L/H) and heater set at 25 °C (25w Aquarium Heater, Boyu, China) and was allocated to one of the following four enrichment treatments at random (*n* = 5): the control (bare tank), 2 cm deep natural gravel substrate (2–4 mm), 2 cm deep sand substrate (sand mix; silica-based), or two plastic plants (20 cm high). The tanks were assigned a number for reference during the statistical analyses (tank number). To prevent any initial microbial contamination and to ensure all tanks were at the same starting point, the gravel and sand were washed, autoclaved and dried, and the plastic plants were soaked in the commercial disinfectant Virkon^®^, (Pittsburgh, PA, USA) before rinsing with distilled water. Tanks, heaters and filters were washed with Virkon^®^ before use. Once the tanks were set up, the commercial products API Stress Coat^®^ (water conditioner; Pittsburgh, PA, USA) and Quick Start^®^ (nitrifying bacteria; Pittsburgh, PA, USA) were added following manufacturer guidelines and the tanks were fed daily with a pinch of Aquarian^®^ Tropical flake (Pittsburgh, PA, USA) and API^®^ Tropical Mini pellets (Pittsburgh, PA, USA) to ensure a source of nitrogen to aid tank cycling. The amount added to the first tank was weighed and replicated across tanks to ensure they were fed equally. Tanks were left for 2 weeks before stocking fish, during which time water quality was tested daily to ensure the filter had developed sufficiently and that the water parameters were appropriate. During this time, any water lost by evaporation was topped up with water from a large container of heated (25 °C), charcoal-filtered tap water that had been left to stand for at least 48 h. Tanks were held under ambient lighting conditions, with natural daylight entering the room. In addition, overhead room lighting (370 ± 15 lux; mean ± SEM) was turned on between 09:00 and 17:00 Monday to Friday.

At the start of the experiment, each tank was stocked with 12 mixed sex zebrafish (*Danio rerio*; ~4 months old) purchased from a local aquatics wholesaler. The experiment consisted of two separate, seven-week periods to account for potential differences in water quality related to the season. The first period was late July to early September (notionally UK ‘summer’) and the other mid-December to mid-February (notionally UK ‘winter’). Between the two trials, tanks were completely drained, cleaned, set up as above and stocked with new fish. There were no mortalities during the trial periods. Following the introduction of fishes, water changes (50%) were carried out weekly and water was replaced with filtered tap water as mentioned above. Water was removed using a siphon cleaned with Virkon^®^, along with any food or faecal material that was visible on the tank bottom. Zebrafish were fed twice daily with a mixture of Aquarian^®^ Tropical flake and API^®^ Tropical Mini pellets. On the first day, the mass of food added to a tank until the fish reached satiation was determined and the same amount then replicated across all tanks to ensure all tanks were fed equally. Fish were rehomed at the end of the experiment.

### 2.1. Water Sample Collection and Analysis

Water samples were collected weekly immediately prior to water changes, from each tank over the 7-week period, starting on the day the fish were introduced to the tanks (week 0). A water sample was also taken from the large container with charcoal-filtered tap water that was used for water changes. Water was collected in a sterile beaker for immediate measurement of temperature (°C), pH (IntelliCALTM pH probe, Loveland, CO, USA) and dissolved oxygen (LDO101 probe with HQ30d meter, Loveland, CO, US). Water samples were then collected in two 50 mL homopolymer polypropylene sample tubes; one tube was frozen immediately at −20 °C for later analysis of ammonia and the second was filtered (FilterMate^TM^ (Kansas City, MO, USA) 0.45 μm PDVF with PTFE prefilter) and a subsample (100 μL) was added to 10 mL of pre-acidified ultrapure water (1 µL ICPMS grade nitric acid) and stored at 4 °C for later analysis of Al, Ca^2+^, Cu, K^+^, Mg^2+^, Na^+^ and Pb (Perkin Elmer Avio-500 ICP-OES, Waltham, MA, USA). A multi-element standard (Fisher Scientific, Hampton, NH, USA, product code 1009-2633) was used for calibration and the wavelengths used were as follows: Al (396.153 nm), Ca^2+^ (317 nm), Cu (324.752 nm), K^+^ (766 nm), Mg^2+^ (285 nm), Na^+^ (589.592 nm), Pb (220.353 nm). Al, Cu and Pb were all below the detection levels. Total ammonia nitrogen (TAN) was quantified using a microplate colourimetric procedure [31] with standards and samples run in triplicate. A Dionex (Sunnyvale, CA, USA) ICS-1100 ion chromatography system was used to determine nitrate (NO_3_^−^) concentration. The conditions were as follows: column: Dionex IonPac AS14a (4 mm × 250 mm); delivery speed: 4 mL min^−1^; delay volume: 125 μL; flow rate: 1 mL min^−1^; eluents: 0.25 mM sodium carbonate (Na_2_CO_3_) and 0.25 mM sodium bicarbonate (NaHCO_3_) in a ratio of 8:1. Total run time was 20 min.

On the day the fish were introduced to the tank (week 0), an additional water sample was collected from each tank in a sterile falcon tube (50 mL) and stored at −20 °C for microbial analysis. A sample for microbial analysis was also taken from the large container with heated, charcoal-filtered tap water. To ensure that any microbes attached within the tank were sampled, in the control (bare) tanks and those with plastic plants, a sterile swab was wiped around the inside of the tank and on the plastic plants for a period of 5 s. The swab was then stirred around in the water sample for 10 s. In the tanks containing substrate, ~1 g of substrate was removed, along with the water sample and the water/substrate sample inverted for 10 s. This sampling procedure was repeated on weeks 1, 3, 5 and 7 of the 7-week study. For microbial analysis, samples were centrifuged for 5 min at 5000× *g* and filtered through a membrane filter (0.45 μm pore size). DNA was then isolated from the sample water (PureLink Microbiome DNA Purification kit, ThermoFisher, Waltham, MA, USA) and PCR carried out with *Pseudomonas*-specific primers (forward 5′-GACGGGTGAGTAATGCCTA-3′; reverse 5′-CACTGGTGTTCCTTCCTATA-3′) and recorded as present or absent. For PCR, initial denaturation was 95 °C for 2 min and cycling conditions for all primers were 35 cycles of 95 °C for 30 s, 63 °C for 45 s and 72 °C for 1 min. To ensure complete extension, the cycling conditions ended with a temperature rise to 72 °C for 10 min. Then, 20 μL of each sample/negative control was analysed by gel electrophoresis on an ethidium bromide stained 2% agarose gel.

### 2.2. Statistical Analyses

Statistical analyses were carried out based on [32] using R ver. 3.6.1. [31]. Error structures, including homogeneity of variances for linear and generalised linear models, were determined by visual inspection of the residuals and QQ plots [32,33]. For ease of reporting, the data associated with the first 7-week trial are referred to as ‘summer’ and the later trial as ‘winter’; the periods themselves do not necessarily represent the season in its entirety. The response variables were fitted to a generalised linear mixed model (GLMM) with treatment, season and week as fixed factors, with the inclusion of the interactions of treatment × season and season × week and tank number as a random factor, using the lme function from the nlme package [34]. Due to the lack of variation within the weeks of sample collection, the variable “week” was not included in the model for *Pseudomonas* spp. presence. For non-normally distributed residuals, data were log or square root transformed and the most appropriate transformation determined by visual inspection of the data. The transformed data were modelled, and residual distribution was inspected as described above. Fixed explanatory variables that did not significantly improve the fit of the model based on delta AIC in stepAIC were removed from the model [35]. Percentage data were expressed as proportions and a binomial GLMM was carried out with the fixed and random variables as described above. Model simplification was performed using delta AIC as previously described and *p* values for fixed terms derived from Chi-square log-likelihood test. The significance of the covariates was tested by ANOVA (using the Anova function in the car package) [33,36]. Pair-wise post-hoc Tukey tests were carried out between the significant interactions using the emmeans package [37]. Figures were created using the ggplot2 package [38]. Significance was set at *p* < 0.05.

## 3. Results

**Figure 1:**
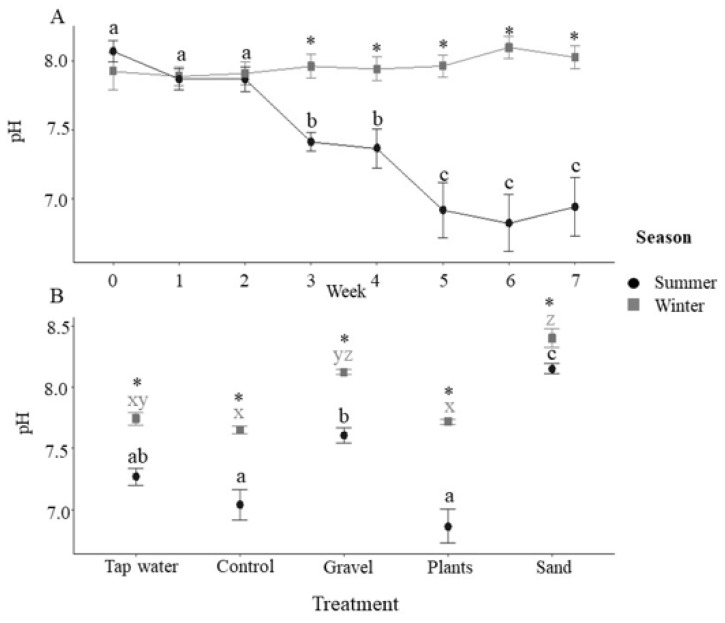
Tank pH. (**A**) Tank pH during summer and winter for each week of the 7-week experimental period (*n* = 20 tanks at each time point combined across treatments). Asterisks indicate significance between season within a time point and different letters indicate significance between weeks within the same season (post-hoc Tukey *p* < 0.05). (**B**) Tank pH by enrichment method with incoming tap water values also presented (tap water: *n* = 7; for control, gravel, plants, sand, *n* = 35 i.e., 5 tanks × 7 weeks with data combined across time). Asterisks indicate a significance between seasons within a treatment and different letters indicate significance between treatments within the same season (post-hoc Tukey *p* < 0.05). All data are means ± SEM.

The majority of water quality parameters measured were significantly affected by both enrichment treatment and season (see Table 1 for full statistical results). The pH in the tanks was significantly lower during the summer months, reflecting a decrease in pH in the incoming water, and in both seasons, it was significantly lower in the control and plant enrichment tanks than in the gravel and sand enrichment treatments (Figure 1B). In the summer, the pH decreased during the latter part of the study, but remained stable throughout the winter (Figure 1A).

**Figure 2:**
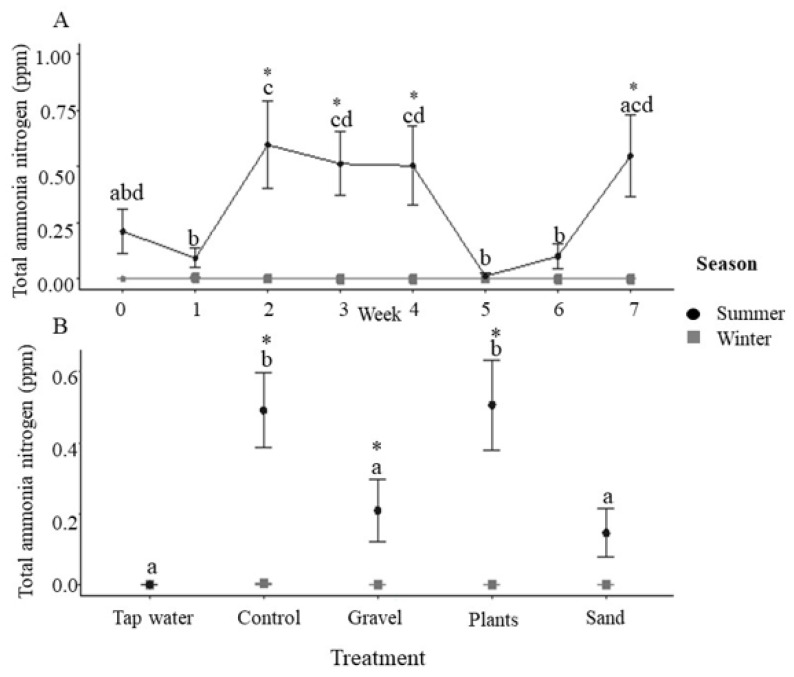
Tank concentrations of total ammonia nitrogen. (**A**) Tank total ammonia nitrogen (TAN; ppm) during summer and winter for each week of the 7-week experimental period (*n* = 20 tanks at each time point combined across treatments). Asterisks indicate significance between season within a time point and different letters indicate significance between weeks within the same season (post-hoc Tukey *p* < 0.05). (**B**) Tank TAN (ppm) by enrichment method with incoming tap water values also presented (tap water: *n* = 7; for control, gravel, plants, sand, *n* = 35 i.e., 5 tanks × 7 weeks with data combined across time). Asterisks indicate significance between seasons within a treatment and different letters indicate significance between treatments within the same season (post-hoc Tukey *p* < 0.05). All data are means ± SEM.

**Figure 3:**
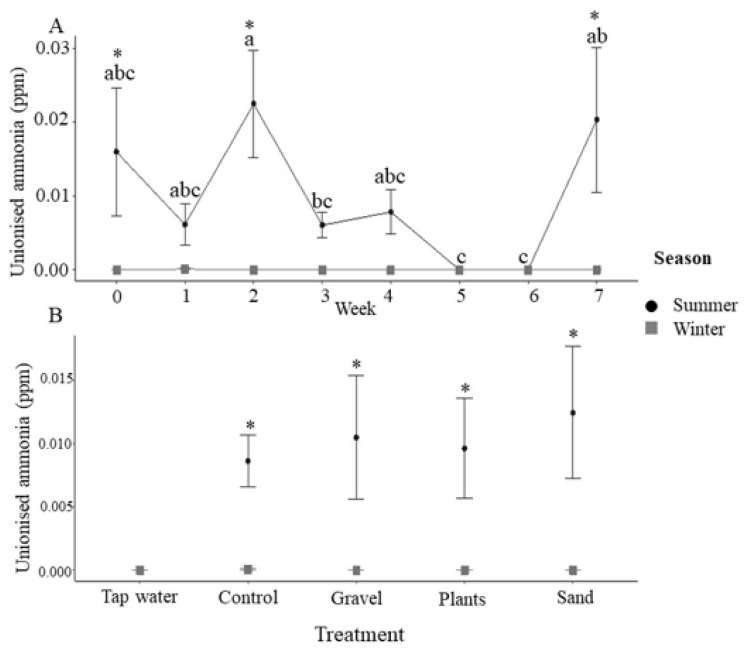
Tank concentrations of unionised ammonia. (**A**) Unionised ammonia (ppm) during summer and winter for each week of the 7-week experimental period (*n* = 20 tanks at each time point combined across treatments). Asterisks indicate significance between season within a time point and different letters indicate significance between weeks within the same season (post-hoc Tukey *p* < 0.05). (**B**) Unionised ammonia (ppm) by enrichment method with incoming tap water values also presented (tap water: *n* = 7; for control, gravel, plants, sand, *n* = 35 i.e., 5 tanks × 7 weeks with data combined across time). Asterisks indicate significance between seasons within a treatment (post-hoc Tukey *p* < 0.05). All data are means ± SEM.

Incoming water was consistently held at 25 °C and despite the heaters present in each tank, a small but significant elevation in temperature was observed during the summer, which did not vary with enrichment treatment (Supplementary Figure S1). Dissolved oxygen was variable throughout the experiment, but with no discernible patterns generated by season or enrichment treatments (Supplementary Figure S2). Total ammonia nitrogen (TAN) remained below the detection levels in the winter (Figure 2) and in summer, it varied with time (Figure 2A), with significantly higher concentrations in the bare tanks and plant enriched tanks than in the incoming water, gravel and sand enrichment treatments (Figure 2B). When adjusted for pH and temperature, unionised ammonia remained significantly lower in the winter (Figure 3A), but the effect of enrichment treatment observed with TAN was lost, with no significant effect of enrichment (Figure 3B).

**Figure 4:**
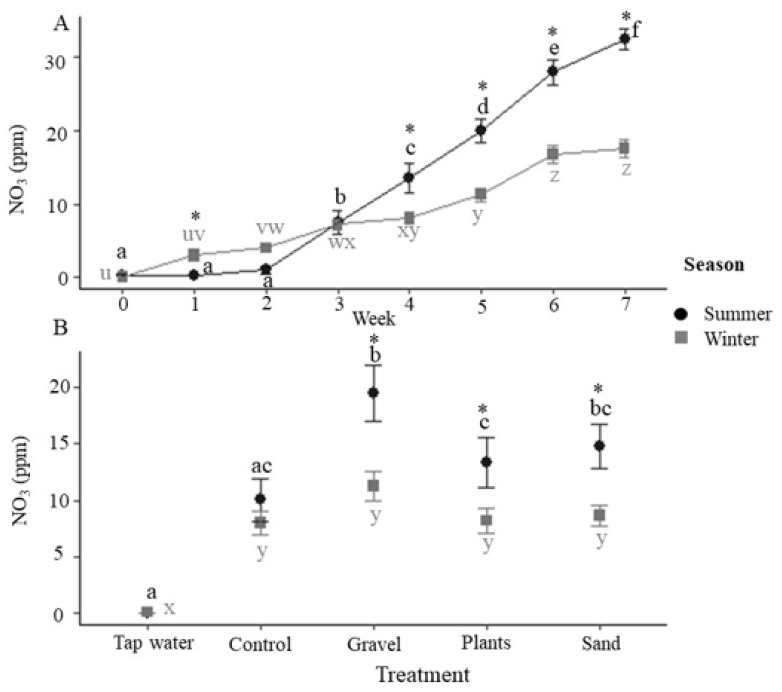
Tank concentrations of nitrate. (**A**) Nitrate (ppm) during summer and winter for each week of the 7-week experimental period (*n* = 20 tanks at each time point combined across treatments). Asterisks indicate significance between seasons within a time point and different letters indicate significance between weeks within the same season (post-hoc Tukey *p* < 0.05). (**B**) Nitrate (ppm) by enrichment method with incoming tap water values also presented (tap water: *n* = 7; for control, gravel, plants, sand, *n* = 35 i.e., 5 tanks × 7 weeks with data combined across time). Asterisks indicate significance between seasons within a treatment and different letters indicate significance between treatments within the same season (post-hoc Tukey *p* < 0.05). All data are means ± SEM.

Nitrate (NO_3_^−^) increased over time in both seasons (Figure 4A) and while there was no effect of the substrate in the winter, during the summer, there were significantly higher concentrations in the gravel tanks than in the incoming water, control and plant enriched tanks (Figure 4B). Nitrate also appeared to be elevated in the sand substrate tanks, which was the intermediate between the control and gravel tank concentrations but not significantly different from either.

**Figure 5:**
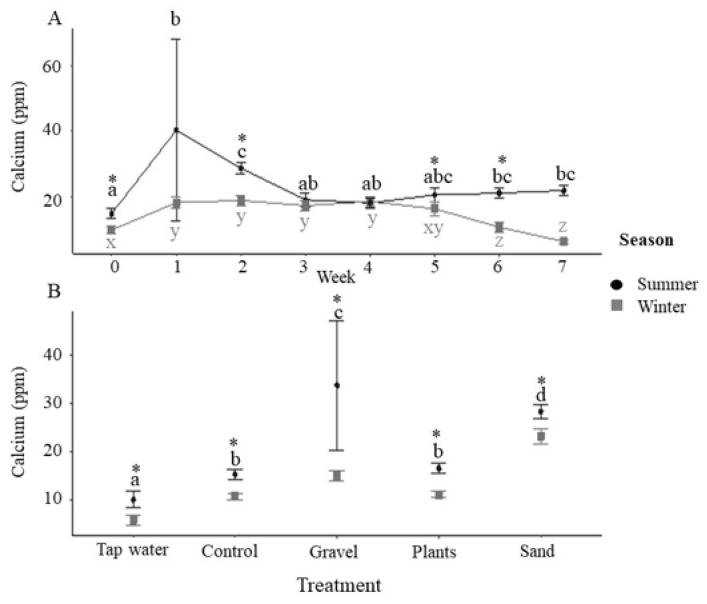
Tank concentrations of calcium. (**A**) Calcium concentrations (ppm) during summer and winter for each week of the 7-week experimental period (*n* = 20 tanks at each time point combined across treatments). Asterisks indicate significance between seasons within a time point and different letters indicate significance between weeks within the same season (post-hoc Tukey *p* < 0.05). (**B**) Calcium concentration (ppm) by enrichment method with incoming tap water values also presented (tap water: *n* = 7; for control, gravel, plants, sand, *n* = 35 i.e., 5 tanks × 7 weeks with data combined across time). Asterisks indicate significance between seasons within a treatment and different letters indicate significance between treatments within the same season (post-hoc Tukey *p* < 0.05). All data are means ± SEM.

Calcium concentrations in the water were significantly higher in the summer than in the winter (Figure 5A). Calcium concentrations were also affected by enrichment treatment, being higher with the gravel and sand enrichment than in the bare tanks and those with plastic plants as enrichment (Figure 5B). Calcium concentrations were lower in the incoming tap water than all the tanks.

**Figure 6:**
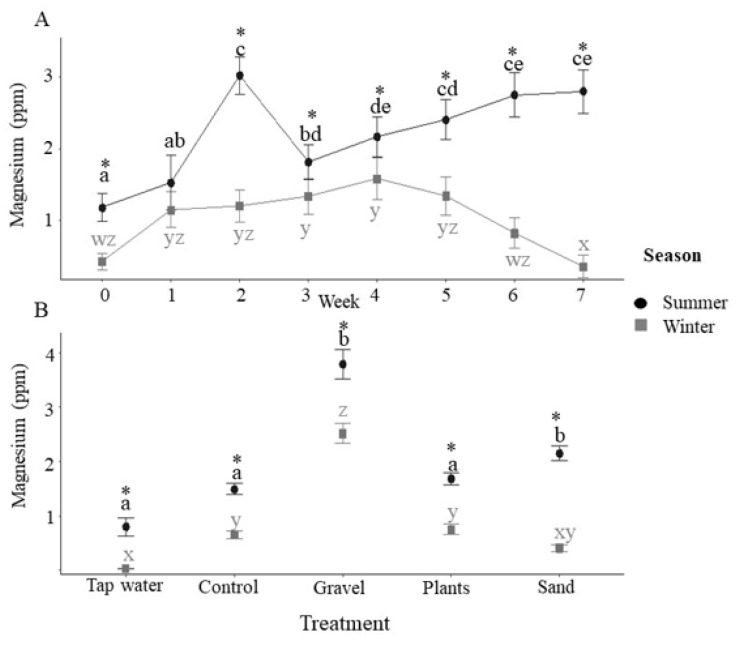
Tank concentrations of magnesium. (**A**) Magnesium concentrations (ppm) during summer and winter for each week of the 7-week experimental period (*n* = 20 tanks at each time point combined across treatments). Asterisks indicate significance between seasons within a time point and different letters indicate significance between weeks within the same season (post-hoc Tukey *p* < 0.05). (**B**) Magnesium concentration (ppm) by enrichment method with incoming tap water values also presented (tap water: *n* = 7; for control, gravel, plants, sand, *n* = 35 i.e., 5 tanks × 7 weeks with data combined across time). Asterisks indicate significance between seasons within a treatment and different letters indicate significance between treatments within the same season (post-hoc Tukey *p* < 0.05). All data are means ± SEM.

Magnesium concentrations showed a similar pattern to calcium concentrations and were significantly higher in the summer than in the winter (Figure 6A) and elevated in the tanks containing sand and gravel as enrichment (Figure 6B). Overall, sodium and potassium concentrations were significantly higher in the tank water in the summer than in the winter (Supplementary Figures S3 and S4), although there was no significant effect of season on the concentrations of these ions in the incoming water. Enrichment type had no significant effect on either sodium or potassium concentrations.

**Figure 7:**
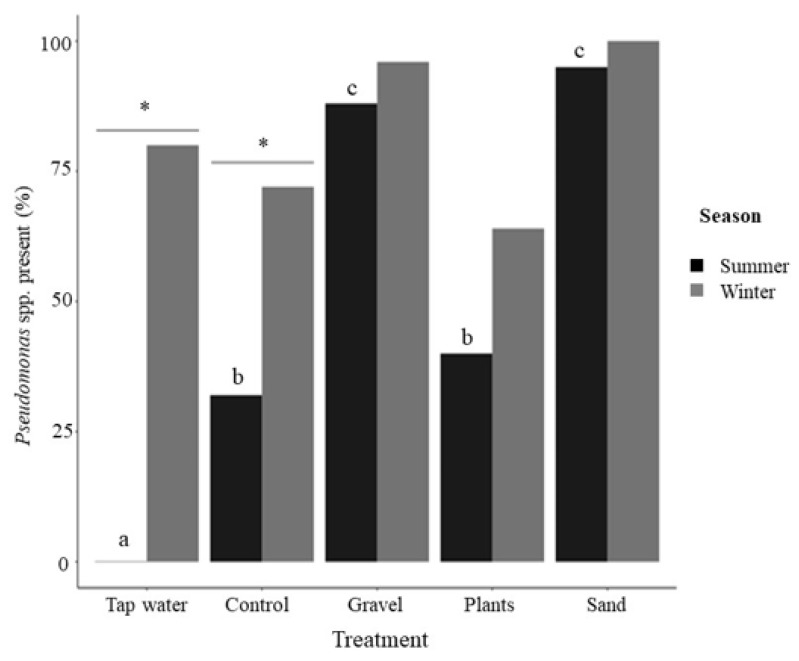
Presence of *Pseudomonas* in tanks. Percent of positive *Pseudomonas* spp. samples by enrichment treatment and season. Asterisks indicate significance between seasons within a treatment (post-hoc Tukey *p* < 0.05). Different letters indicate significance between enrichment treatments within a season (post-hoc Tukey *p* < 0.05). Data are means ±SEM, tap water: *n* = 7; for control, gravel, plants, sand, *n* = 35 (i.e., 5 tanks × 7 weeks with data combined across time).

Both treatment (*X^2^* = 9.724, df = 1, *p* = 0.002) and season (*X^2^* = 38.076, df = 4, *p* < 0.0001; season × treatment: *X^2^* = 67.245, df = 5, *p* < 0.0001) had a significant effect on the presence of *Pseudomonas* spp. (Figure 7). In the summer, a greater number of samples tested positive for the presence of *Pseudomonas* spp. in the tanks containing gravel and sand, compared with the control tanks and those with plastic plants as enrichment. In the winter, significantly more samples tested positive in the incoming water than in the summer, which may have masked any significant effect of enrichment treatment.

## 4. Discussion

In the present study, we considered whether environmental enrichment and time of year affected water quality in tropical freshwater home aquaria. Both gravel and sand substrate increased the pH of tank water significantly compared to tanks without substrate. The sand was made from silicate aggregates; both sand and gravel were commercially available aquarium substrates sold as products that do not influence pH. Substrates were thoroughly washed, autoclaved and dried prior to the start of the experiment, so the increased pH was likely driven by the dissolution of mineral phases, such as carbonates and feldspars from the gravel and sand, throughout the course of the experiment. The effect of substrate on pH was present in both trials, and while the pH remained stable during the winter, a decrease in overall pH was observed with time over the summer experimental period, which could have been driven by a variety of different factors, including temperature. Type of substrate is likely to influence the effects on pH and in this experiment, sand resulted in a significantly elevated pH compared to gravel in the summer, but in the winter, there was no significant difference between the two substrates. A previous experiment using glass chips as substrate found no significant effect of pH [12].

In addition to differences in pH, substrate treatments were also associated with increased concentrations of calcium and magnesium. Potentially, these elements represent leaching from the substrate and may explain the more alkaline pH associated with substrate treatments. Concentrations of magnesium and calcium were higher in the incoming water in the summer, likely explained by lower summer pH, leading to greater dissolution of partially reactive minerals. These differences may also reflect complex differences in the microbial/microalgal community, driven by seasonal differences in water treatment or ambient light [39]. Higher concentrations of calcium and magnesium are consistent with a previous study on the water quality of four streams in the Cairngorm Mountains of Scotland in which weathering-derived elements, such as calcium and magnesium, were found to be higher in the summer [40]. Several other studies have found that water quality changes with season in Scotland, owing to the hydrological processes that route precipitation [29,40,41]. Sodium and potassium were also higher in tank water in the summer, but no significant differences were noted in the incoming tap water, suggesting that this was driven by fish presence rather than seasonal variations in water quality. It is possible that higher temperatures and higher concentrations of nitrogenous waste products during the summer period increased the efflux of these ions from the fish, as stress is known to elicit increased excretion of sodium [42].

Total ammonia nitrogen (TAN) was low in the incoming water in both trials and remained below detection in the winter, but was elevated in all treatments compared to the incoming tap water in the summer. Gravel and sand enrichment reduced TAN compared to the control and plants, but had elevated concentrations of nitrate. This is similar to a study on gilthead seabream, *Sparus aurata*, where the tanks containing glass chips as substrate had higher levels of nitrite compared with the bare tanks [12]. Lower concentrations of TAN, coupled with higher levels of nitrate, could potentially be due to the larger surface area that the gravel and sand particles provide for the growth of nitrification bacteria, which may be responsible for the oxidation of ammonia to nitrite and then nitrate. A range of nitrifying taxa have been identified in freshwater aquaculture biofilters, including *Nitrospira* spp., ammonia-oxidising archaea (AOA) and *Nitrotoga* [43], with *Nitrosomonas* and *Nitrobacter* no longer believed to predominate in freshwater aquarium systems [44]. Sand-associated taxa often include nitrifiers [43], and as the microbial community associated with the water and substrate are likely to be different [45], the presence of substrate could provide an enhanced opportunity for bacteria that oxidise ammonia.

Ammonia is excreted across the gills of fishes and constitutes their main nitrogenous waste product. TAN comprises un-ionised (NH_3_) and ionised ammonia (NH_4_^+^), with the former being particularly toxic to fishes [46]. Ammonia toxicity (measured as the LC_50_) occurs, on average, at 2.79 mg NH_3_ L^−1^ in freshwater fishes and 1.86 mg NH_3_ L^−1^ in seawater fishes [47]; the concentrations of ammonia in the present study always stayed well below toxic levels. The exact composition of TAN is dependent upon temperature and pH [48], where both increasing temperature and increasing pH result in increased levels of unionised ammonia. In the present study, the temperature in the tanks was slightly, but significantly, higher in the summer. The significantly lower pH found in the bare tanks and tanks with plastic plants, combined with the higher concentrations of TAN summer, led to no significant differences in unionised ammonia when compared across treatments. Therefore, although the enrichment treatment appears to affect water quality, the effects on pH and TAN effectively cancelled each other out in relation to potential ammonia toxicity. While the NO_3_^−^ concentrations remained below the levels of 50 mg L^−1^, which have been observed to lead to poor growth, lethargy, anorexia and opportunistic infections, [49], increased nitrification in the presence of gravel, and potentially sand substrates, could lead to problems with nitrate toxicity.

Although tap water is treated by disinfection with chlorine at water treatment plants, it fails to kill all bacteria [50,51] and *Pseudomonas* has previously been found in drinking water [52]. In the present study, *Pseudomonas* was only found in the incoming tap water in the winter; a study on the presence of *Aeromonas* in treated public drinking water in north-east Scotland [53] identified a correlation with increased recovery of *Aeromonas* and increased precipitation. In summer, there was a clear impact of substrate on *Pseudomonas,* with both substrate treatments demonstrating a greater presence of *Pseudomonas*. A similar pattern was observed in winter but during this period, the high presence of *Pseudomonas* in the incoming tap water obscured any effects of treatment. *Pseudomonas* spp. was chosen as an indicative bacterium to understand the effects of substrate presence on bacteria in general. The genus *Pseudomonas* contains species and strains that can be both pathogenic to fishes [54] but additionally, several species of *Pseudomonas* have been found to have nitrification activity [55] or to improve fish survival [56]. As discussed above, substrate enrichment may have the capacity to increase nitrifying potential of the bacterial community, but may also provide an increased surface area for obligate or opportunistic pathogens. Alternatively, more complex microbial communities associated with substrate enrichment may make it more difficult for pathogens to establish. Fishes that experience stress can be more susceptible to pathogenic microbes, and disease due to microbial pathogens is a widespread problem in the ornamental fish industry [22,30,57,58]. Our understanding of the microbiome within ornamental fish tanks remains minimal and is a key area for future research.

## 5. Conclusions

In conclusion, the type of environmental enrichment used within freshwater home aquaria significantly altered water chemistry and *Pseudomonas* spp. presence, with some of these effects varying with the time of year. Overall, the addition of substrate as enrichment resulted in increased pH and nitrate and a greater presence of bacteria. Studies that consider water quality parameters in relation to fish welfare and the use of enrichment need to consider the effects of season, as this can have a confounding effect. Further research is clearly needed to understand how substrate alters the tank microbiome both in terms of beneficial and also pathogenic organisms, and the subsequent implications for fish welfare.

## Figures and Tables

**Table 1:** Full statistical results for water quality. ANOVA results showing F values, degrees of freedom and *p* values for all water parameters tested as a function of enrichment treatment, season and week with interactions between factors. Significant *p* values are indicated in bold.

Parameter	Treatment	Season	Week	Treatment × Season	Season × Week
Oxygen	F_3,16_ = 0.5; *p* = 0.6663	F_1,285_ = 2.6; *p* = 0.107	F_7,285_ = 25.8; *p* < **0.0001**	F_3,282_ = 2.0; *p* = 0.119	F_7,285_ = 32.1; *p* < **0.0001**
Temperature	F_3,16_ = 0.03; *p* = 0.994	F_1,281_ = 114.7; *p* < **0.0001**	F_7,281_ = 2.9; *p* = **0.0050**	F_3,281_ = 6.9; *p* = **0.0003**	F_7,281_ = 7.4; *p* < **0.0001**
pH	F_4,16_ = 75.1; *p* < **0.0001**	F_1,295_ = 176.3; *p* < **0.0001**	F_7,295_ = 14.3; *p* < **0.0001**	F_4,295_ = 8.8; *p* < **0.0001**	F_7,295_ = 22.3; *p* < **0.0001**
TAN ^1^	F_4,16_ = 5.1; *p* = **0.0076**	F_1,293_ = 70.6; *p* < **0.0001**	F_7,293_ = 4.1; *p* = **0.0003**	F_7,293_ = 5.0; *p* = **0.0006**	F_4,293_ = 4.2; *p* = **0.0002**
Unionised ammonia	F_4,16_ = 0; *p* = 0.7896	F_1,297_ = 27; *p* < **0.0001**	F_7,297_ = 3; *p* = **0.0137**	F_4,293_ = 0.0; *p* = 0.82	F_7,297_ = 3.0; *p* = **0.012**
Nitrate	F_4,16_ = 14.9; *p* < **0.0001**	F_1,278_ = 134.7; *p* < **0.0001**	F_7,278_ = 219.4; *p* < **0.0001**	F_4,278_ = 5.1; *p* < **0.0001**	F_7,278_ = 25.7; *p* < **0.0001**
Calcium	F_4,16_ = 40.6; *p* < **0.0001**	F_1,296_ = 57.5; *p* < **0.0001**	F_7,296_ = 12.3; *p* < **0.0001**	F_4,292_ = 0.6; *p* = 0.6498	F_7,296_ = 10.5; *p* < **0.0001**
Potassium	F_4,16_ = 4.945; *p* = **0.0087**	F_1,292_ = 248.9; *p* < **0.0001**	F_7,292_ = 110.3; *p* < **0.0001**	F_4,292_ = 5.5; *p* = **0.0003**	F_7,292_ = 32.1; *p* < **0.0001**
Magnesium	F_4,16_ = 62.3; *p* < **0.0001**	F_1,292_ = 307.6; *p* < **0.0001**	F_7,292_ = 18.4; *p* < **0.0001**	F_4,292_ = 10.1; *p* < **0.0001**	F_7,292_ = 15.3; *p* < **0.0001**
Sodium	F_4,16_ = 8.5; *p* = **0.0007**	F_1,292_ = 1144.5; *p* < **0.0001**	F_7,292_ = 73.6; *p* < **0.0001**	F_4,292_ = 7.5; *p* < **0.0001**	F_7,292_ = 35.8; *p* < **0.0001**

^1^ TAN = total ammonia nitrogen.

## Data Availability

Data from this work will be made available in a publicly accessible repository upon acceptance of the manuscript.

## Supplementary Materials

The following supporting information can be downloaded at: https://www.mdpi.com/article/10.3390/ani12192679/s1, Figure S1: Tank temperature; Figure S2: Tank oxygen; Figure S3: Tank concentrations of potassium; Figure S4: Tank concentrations of sodium.
