# Supplementary material for: The Effect of Substrate on Water Quality in Ornamental Fish Tanks
Source: Animals (Basel). 2022 Oct 5;12(19):2679. doi: 10.3390/ani12192679 (PMC9558538; doi:10.3390/ani12192679)
Supplement: Supplementary file 1 [file animals-12-02679-s001.zip › animals-1934968-supplementary.pdf]

animals

MDPI

Supplementary Material

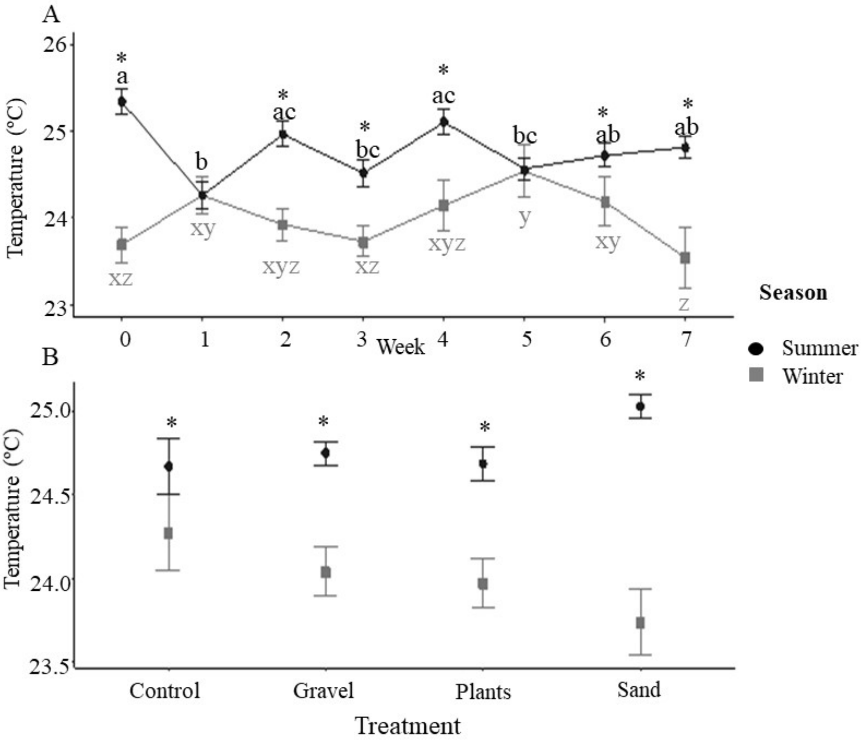

Figure S1. Tank temperature. (**a**) Temperature (° C) during summer and winter for each week of the 7-week experimental period (n=20 tanks at each time point combined across treatments). Asterisks indicate significance between season within a time point and different letters indicate significance between weeks within the same season (post-hoc Tukey $P<0.05$). (**b**) Temperature (° C) by enrichment method with incoming tap water values also presented (tap water: n=7; for control, gravel, plants, sand, n=35 i.e. 5 tanks x 7 weeks with data combined across time). Asterisks indicate significance between season within a treatment (post-hoc Tukey $P<0.05$). All data are means ± SEM.

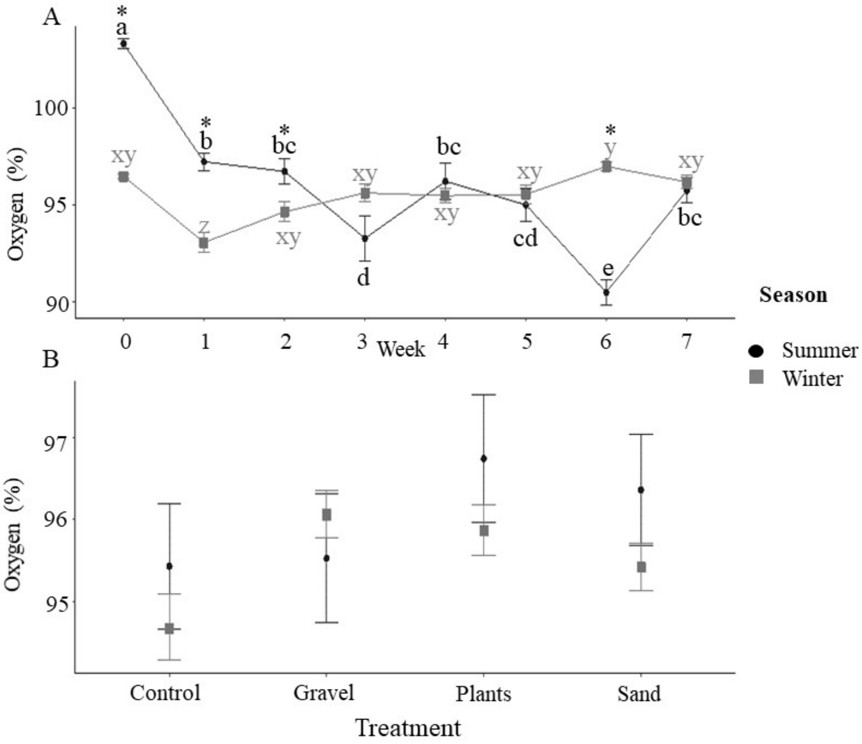

Figure S2. Tank dissolved oxygen. (a) Dissolved oxygen (%) during summer and winter for each week of the 7-week experimental period (n=20 tanks at each time point combined across treatments). Asterisks indicate significance between season within a time point and different letters indicate significance between weeks within the same season (post-hoc Tukey P<0.05). (**b**) Dissolved oxygen (%) by enrichment method with incoming tap water values also presented (tap water: n=7; for control, gravel, plants, sand, n=35 i.e. 5 tanks x 7 weeks with data combined across time). All data are means ± SEM.

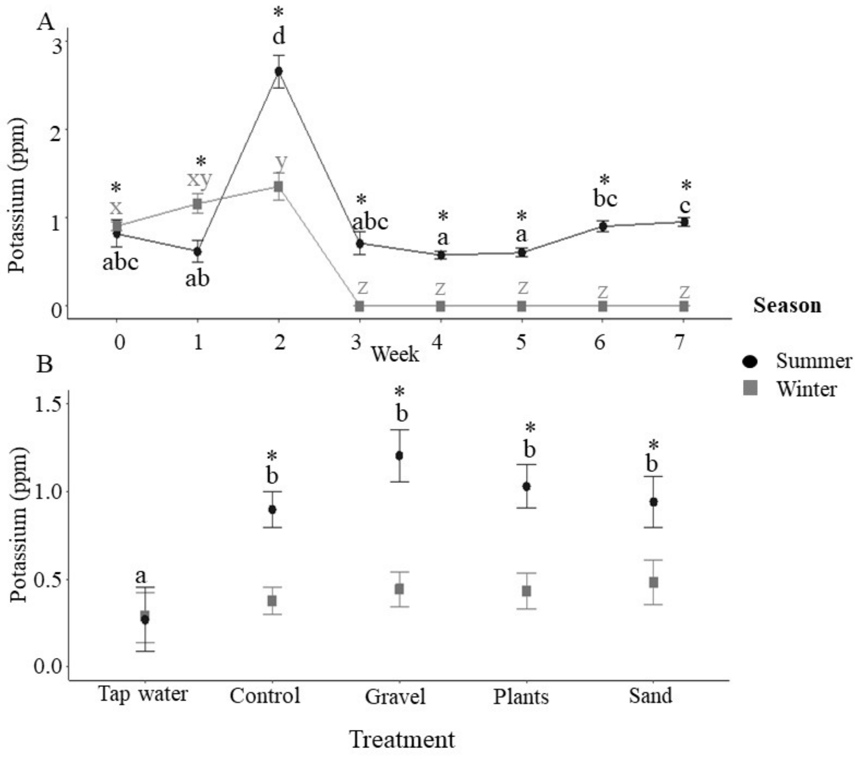

Figure S3. Tank concentrations of potassium. (**a**) Potassium concentrations (ppm) during summer and winter for each week of the 7-week experimental period (n=20 tanks at each time point combined across treatments). Asterisks indicate significance between season within a time point and different letters indicate significance between weeks within the same season (post-hoc Tukey P<0.05). (**b**) Potassium concentration (ppm) by enrichment method with incoming tap water values also presented (tap water: n=7; for control, gravel, plants, sand, n=35 i.e. 5 tanks x 7 weeks with data combined across time). Asterisks indicate significance between season within a treatment and different letters indicate significance between treatments within the same season (post-hoc Tukey P<0.05). All data are means ± SEM.

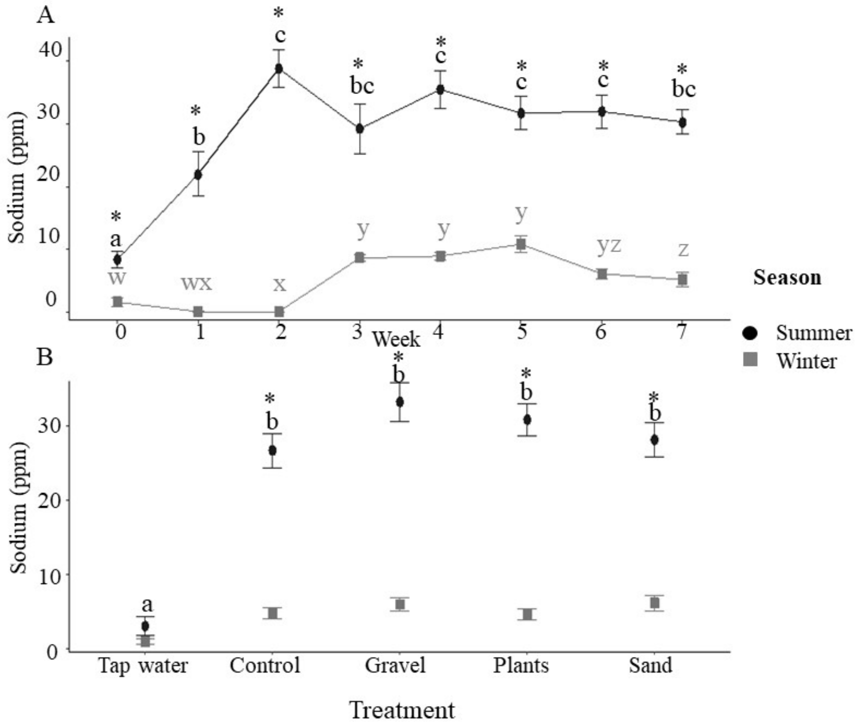

Figure S4. Tank concentrations of sodium. (**a**) Sodium concentrations (ppm) during summer and winter for each week of the 7-week experimental period (n=20 tanks at each time point combined across treatments). Asterisks indicate significance between season within a time point and different letters indicate significance between weeks within the same season (post-hoc Tukey P<0.05). (**b**) Sodium concentration (ppm) by enrichment method with incoming tap water values also presented (tap water: n=7; for control, gravel, plants, sand, n=35 i.e. 5 tanks x 7 weeks with data combined across time). Asterisks indicate significance between season within a treatment and different letters indicate significance between treatments within the same season (post-hoc Tukey P<0.05). All data are means ± SEM.
